# Using natural language processing to automatically classify written self-reported narratives by patients with migraine or cluster headache

**DOI:** 10.1186/s10194-022-01490-0

**Published:** 2022-09-30

**Authors:** Nicolas Vandenbussche, Cynthia Van Hee, Véronique Hoste, Koen Paemeleire

**Affiliations:** 1 Department of Neurology, Ghent University Hospital, Corneel Heymanslaan 10, 9000 Ghent, Belgium; 2 Department of Basic and Applied Medical Sciences, Faculty of Medicine and Health Sciences, Ghent University, Corneel Heymanslaan 10, 9000 Ghent, Belgium; 3 LT3 – Language and Translation Technology Team, Department of Translation, Interpreting and Communication, Faculty of Arts and Philosophy, Ghent University, Groot-Brittanniëlaan 45, B-9000 Ghent, Belgium

**Keywords:** Migraine, Cluster headache, Natural language processing, Machine learning

## Abstract

**Background:**

Headache medicine is largely based on detailed history taking by physicians analysing patients’ descriptions of headache. Natural language processing (NLP) structures and processes linguistic data into quantifiable units. In this study, we apply these digital techniques on self-reported narratives by patients with headache disorders to research the potential of analysing and automatically classifying human-generated text and information extraction in clinical contexts.

**Methods:**

A prospective cross-sectional clinical trial collected self-reported narratives on headache disorders from participants with either migraine or cluster headache. NLP was applied for the analysis of lexical, semantic and thematic properties of the texts. Machine learning (ML) algorithms were applied to classify the descriptions of headache attacks from individual participants into their correct group (migraine versus cluster headache).

**Results:**

One-hundred and twenty-one patients (81 participants with migraine and 40 participants with cluster headache) provided a self-reported narrative on their headache disorder. Lexical analysis of this text corpus resulted in several specific key words per diagnostic group (cluster headache: *Dutch* (*nl): “oog” | English (en): “eye”*, *nl: “pijn” | en: “pain”* and *nl: “terug” | en: “back/to come back”;* migraine: *nl: “hoofdpijn” | en: “headache”*, *nl: “stress” | en: “stress”* and *nl: “misselijkheid” | en: “nausea”*). Thematic and sentiment analysis of text revealed largely negative sentiment in texts by both patients with migraine and cluster headache. Logistic regression and support vector machine algorithms with different feature groups performed best for the classification of attack descriptions (with F1-scores for detecting cluster headache varying between 0.82 and 0.86) compared to naïve Bayes classifiers.

**Conclusions:**

Differences in lexical choices between patients with migraine and cluster headache are detected with NLP and are congruent with domain expert knowledge of the disorders. Our research shows that ML algorithms have potential to classify patients’ self-reported narratives of migraine or cluster headache with good performance. NLP shows its capability to discern relevant linguistic aspects in narratives from patients with different headache disorders and demonstrates relevance in clinical information extraction. The potential benefits on the classification performance of larger datasets and neural NLP methods can be investigated in the future.

**Trial registration:**

This study was registered with clinicaltrials.gov with ID NCT05377437*.*

**Supplementary Information:**

The online version contains supplementary material available at 10.1186/s10194-022-01490-0.

## Background

Headache disorders are amongst the most prevalent medical conditions worldwide. The Global Burden of Disease study 2016 designated migraine as the second most disabling condition worldwide [1]. It is estimated that almost three billion people worldwide suffer from a headache disorder, with over one billion people suffering from migraine [2]. The International Classification of Headache Disorders, Third Edition (ICHD-3) is the current standard for the diagnosis of headache disorders [3].

In headache medicine, for the largest part, the physician acquires self-reported information about headache characteristics and symptomatology from the patient’s perspective. There are currently no technical investigations or biological markers that allow an accurate and precise diagnosis of primary headache disorders such as tension-type headache, migraine or cluster headache (CH). The physician’s role is to interpret the linguistic data produced by the patient and to analyse the content. A diagnosis is made by applying the different sets of criteria of headache disorders within ICHD-3 to this linguistic information [3].

This diagnostic process is often an intricate step within patient care. Multiple elements need to be assessed to make a diagnosis based on ICHD-3 [3]. Detailed history taking requires training and experience, and may be a time-consuming and labour-intensive process for both physicians and patients [4]. Given the complexity and heterogeneity of headache disorders with a multitude of symptoms occurring before, during and after headache attacks, patients may describe their conditions in various ways [5, 6]. Misdiagnosis or underdiagnosis of headache disorders therefore remain common phenomena. This is especially true for CH patients who may wrongly receive a diagnosis of migraine or other headache disorder [7–11].

In an effort to overcome the complexity of headache diagnostics, our research applies both qualitative and quantitative methods to evaluate the potential of natural language processing (NLP) and machine learning (ML) applications for patients’ narratives of their experiences with headache disorders. NLP is a subfield of computational linguistics which can structure, process and analyse naturally produced language by humans through digital algorithms [12]. ML algorithms are capable of classifying texts into predefined categories.

In this study, we hypothesize that NLP can aid our understanding of patients’ communication about headache disorders. We also hypothesize that ML algorithms processing self-reported narratives on headache, provided here by patients with either migraine or CH, may accurately classify these texts into their correct groups of headache disorders.

## Methods

### Study design and participants

This study was a prospective cross-sectional, monocentric, academic study with patients from a tertiary headache clinic at the Ghent University Hospital, which is located in Flanders, the Dutch-speaking part of Belgium. Recruitment took place between August 27^th^ 2020 and March 11^th^ 2021. During planned outpatient visits, patients were recruited to participate in this study. Participation required that patients had been diagnosed by one of the headache expert neurologists from the Ghent University Hospital according to ICHD-3. Participants were of adult age at the start of the study, had Dutch as their native language and were able to read and provide a self-written text in Dutch. We analysed texts provided by patients with a sole diagnosis of either migraine or CH. There were no other inclusion or exclusion criteria. Due to the exploratory nature of the study, no formal sample size calculation was performed.

### Ethics approval and consent to participate

This study was approved by the Committee for Medical Ethics of Ghent University Hospital (internal ID BC-08263, approved August 18^th^ 2020). Patients were fully informed on all the aspects of the study (duration, procedures, study visit etc.) and gave written informed consent at the beginning of the study. Participants received a pseudonymized code throughout the study. Only physician-researchers had the key to decode the participant if required.

### Study procedures and data collection

One visit in person took place during the study. Participants were fully informed on the study details and provided written informed consent to participate. Participants then gave the researcher their personal e-mail address to which a web-based survey was sent. In the survey, patients were asked to write down a detailed and accurate description of their headache disorder (the full text of the request can be found in Additional File 1 of the supplementary information to this article). Participants had complete freedom to write in their own words and phrases about their headache disorders. There was no limitation on word or sentence count and all topics or themes were allowed. We requested not to write any given and last names or entities (e.g. companies or hospitals) in the text for privacy reasons. When a participant completed the writing task and uploaded the text to the database, the case-report form was completed with demographic and disorder-specific information from electronic medical patient records of the Ghent University Hospital including age, sex and ICHD-3 diagnosis of the participant.

Study data was collected and managed using REDCap (Research Electronic Data Capture) tools hosted at the secure IT environment of the Ghent University Hospital [13, 14]. REDCap is a secure, web-based software platform designed to support data capture for research studies, providing 1) an intuitive interface for validated data capture; 2) audit trails for tracking data manipulation and export procedures; 3) automated export procedures for seamless data downloads to common statistical packages; and 4) procedures for data integration and interoperability with external sources [13, 14]. If the participant did not open the link to the survey in the first e-mail, up to five reminder e-mails were sent automatically on a weekly basis. The written narratives from participants with respective metadata on age, sex and headache diagnosis were exported from the REDCap database and stored in a digital language corpus for further data analysis. For the texts and data on age, sex and headache diagnoses, we found no missing data.

### Demographic and textual characteristics

Demographic characteristics including age and sex are given as mean with standard deviation (SD) and number as well as percentage of female patients respectively.

For the textual analysis, the representation of tokens and types are on the level of words. A token is an instance of a sequence of characters in a particular document that are grouped together as a useful semantic unit for processing. A type is a class containing all tokens with the same character sequence (e.g. “time after time” has 3 tokens and 2 types) [15]. For tokens and types, throughout the article, we provide the original Dutch word and the appropriate English translation of the word with the ISO 639–1 codes for languages: *nl:“…” | en:“…”.*

Total counts of tokens, types and sentences for all corpora and diagnostic subcategories are presented as medians with first and third interquartile values due to the non-normal distribution of the data. Key tokens for the analysis of lexical diversity discerning the two diagnostic categories (migraine and CH) were calculated with chi-square tests [16–19]. All tests documented in this paragraph were performed with R package ‘quanteda’ version 3.2.0 [20].

### Thematic annotation

Manual thematic annotation was performed by the first author (NV, headache neurologist) for every provided text by using NVivo software® (release 1.5, 2021). All texts were read and annotated in seven predefined themes: attack description, burden of disease, comorbidities, technical investigations, triggers, treatment and previous medical history. Annotation was performed at the level of sentences.

For thematic analysis, word token count per theme of all individual full self-narrative texts was calculated within NVivo. The presence of each theme was calculated as proportion of the theme within the full self-narrative text by dividing the sum of the total word token count per theme by the sum of the total word token count of the self-narrative. The results are presented as the median proportion of each theme within a diagnostic category, combined with first and third quartile proportions.

### Machine learning experiments

ML experiments aimed to classify a text in the right diagnostic category. A ML approach is said to learn from experience (E) with respect to some task (T) and some performance measure (P), if its performance on T, as measured by P, improves with experience E [21]. Applied to our dataset, the task T at hand is the binary differentiation between "migraine" and "cluster headache". In order to measure how well the system performs (P), F-measures were used as evaluation metrics. Accuracy presents the fraction of correct predictions, whereas F1-measure is the mean of precision (i.e. positive predictive value) and recall (i.e. sensitivity). The latter metric provides a better understanding of a test’s accuracy in situations where there is class imbalance (e.g. in this study, the number of participants with migraine outweighs the number of participants with CH). E refers to the training data the system is being fed with; the amount of data needed to reach optimal performance is highly dependent on the complexity of the classification task.

For the experiments, we applied the ML algorithms on the parts of the full texts annotated as “attack description”. This is in line with ICHD-3 methodology where semiology of headache attacks is used for the diagnosis of migraine or CH [3]. Due to the small size of the corpus with attack descriptions (*n* = 112), we decided not to split the corpus into separate sets for training, development and testing (often an 80%-10%-10% split is used), but to evaluate experiments within a cross-validation setup instead. Cross-validation is an evaluation method where data are partitioned into a predefined number of segments or “folds” that are used for training and testing in successive rounds. The basic form of cross-validation is “k-fold cross-validation”: the corpus is split into k equally sized folds, after which k iterations of training and testing are performed so that within each iteration a different fold of the data is held out for testing while the remaining k-1 folds are used for training or learning [22]. In data mining and ML, 5 and 10 are common values for k. For our experiments, fivefold cross-validation was preferred over tenfold so as to have a more representative test fold at each iteration (i.e. 1/5 of the 112 instances). All folds were stratified to have a constant label distribution (i.e. 67%-33% for the classes ‘migraine’ and ‘cluster headache’, respectively).

Prior to performing classification experiments, the experimental corpus of attack descriptions was pre-processed, meaning that a number of operations were carried out to transform the data into the most appropriate form for further experimenting. For this study, the pre-processing steps include:

- Manual data cleaning and conversion to UTF-8 character encoding;
- Sentence splitting: automatic detection of sentence boundaries;
- Tokenization: automatic splitting of sentences into tokens;
- (Stop) word filtering: removal of class label mentions and other references that may hint at the class label (e.g. ‘medication’), in addition to stop word removal (i.e. words which typically do not add much meaning to a text but instead ensure the structure of a sentence is appropriate).

For the second and third steps we made use of the LeTs Preprocess pre-processing toolkit for, amongst other languages, Dutch [23]. After tokenization, we removed from the corpus all class label mentions in the instances (i.e. ‘migraine’ and ‘cluster headache’) and other words that hint at or are related to this class label (e.g. specific medication references such as ‘ibuprofen’ or drug brand names). The full list of filtered words can be consulted in Additional File 2. After this step, Dutch stop words were removed from the corpus [12, 24].

We compared the performance of three popular classifiers for binary classification, being naive Bayes (NB), support vector machine (SVM) and logistic regression (LR) [12]. NB is an example of a generative classifier, which builds a model of how a class could generate some input data. Given an unseen test instance, such classifiers return the class that is most likely to have generated the observation. NB is a probabilistic ML algorithm that makes the “bag of words” assumption (i.e. a list of tokens without information on token position or grammar) and the conditional independence assumption (words are conditionally independent of each other given the class) [12]. Discriminative classifiers like SVM and LR are different in that they learn which features from the training data are most useful to discriminate between the different classes observed in the training data. LR is a popular baseline supervised ML algorithm for many classification tasks. In addition, LR classifiers have a close relationship with neural networks, since the latter can be viewed as a series of LR classifiers stacked on top of each other. Such neural networks have recently gained tremendous popularity due to their efficiency and high performance for different kinds of classification tasks in varying domains. One important condition, however, for them to perform adequately, is to have a large dataset available for training, which is why they are less appropriate for the current task. SVM classifiers have been and are still a popular classification algorithm for applications in biomedical and other sciences and text classification tasks like the one in this study [25]. For the experiments described in this section, we compared the performance of these three classifiers in detecting whether patients' descriptions correspond to either a CH diagnosis or a migraine diagnosis. Each classification algorithm has a number of hyperparameters which need to be optimized for the task at hand, such as the kernel for SVM and the C-value, which is a regularization parameter for the SVM and NB algorithms.

The attack descriptions were converted to so-called “feature vectors” (i.e. a n-dimensional vector of numerical properties of the observed data) which were then fed to the three classifiers. Three different feature groups were taken into account:

- word and character n-grams (contiguous sequence of tokens with n ranging between 1 and 3);
- patient metadata features: age and gender information;
- word and character n-grams (with n ranging between 1 and 3) with patient metadata features combined.

The above described classifiers (NB, SVM, LR) combined with three feature groups resulted in nine different experimental setups. As mentioned earlier, we report accuracy and F-measure as the evaluation metrics. Accuracy represents the classifier’s average performance (i.e. calculated on the two classes and by weighting the scores according to the class distribution). As accuracy is biased by class frequency, we also report macro-averaged F1-score which considers both classes as equally important. The experimental scores are presented for the entire dataset and for the CH class only, as this would be the most urgent class to detect in a real-world application. Due to the cross-validation setup, the results we report are averages over our total of k = 5 folds.

It is important to note that, given the small dataset, grid search optimization and classifier evaluation were both done in a nested cross-validation setup with k = 5 as mentioned earlier. Nested cross-validation is an approach to perform hyperparameter optimization (i.e. algorithm-specific settings with high potential to influence the classification performance) and feature selection that attempts to overcome the problem of overfitting. The goal of nested cross-validation is to avoid that test data information ‘leaks’ into the training set because in cross-validation instances are part of a training fold in one iteration, while functioning as test set in another iteration. Concretely, within every outer fold another k-fold cross-validation experiment is carried out to find the classifier's most suited hyperparameter settings given that particular fold. Given the class imbalance in our dataset, grid search optimization was based on the macro-averaged F1-score, meaning that all classes in the training data were given equal weight, irrespective of their original distribution. This way, we forced the system to pay equal attention to the minority CH class compared to the majority migraine class when optimizing the model.

As our experiments were conducted on a small dataset, it is difficult to estimate the true difficulty of the classification task. In fact, many classifiers tend to benefit from more training data so that a more generalizable model can be constructed. We therefore set up an additional experiment using an alternative evaluation method to regular cross-validation, namely leave-one-out cross-validation. The latter is a special case of cross-validation where the number of folds equals the number of instances in the dataset. This means that N-1 (where N is the corpus size) instances are used as training data for the learning algorithm, which makes a prediction for the remaining instance that functions as a single-item test set.

All ML experiments were performed in the Python (NLTK, scikit-learn) and R (base R and packages tidyverse and quanteda) coding environment [20, 26–29].

### Lexicon-based sentiment analysis

Words can be classified as either having a negative or positive sentiment based on annotations in lexicons. Lexicon-based sentiment analysis was performed to analyse the sentiment expressed in the dataset based on a combination of state-of-the-art sentiment lexicons for Dutch (see further). As a pre-processing step, we again removed the words that may hint at the message labels (e.g. ‘cluster headache’ and ‘migraine’ and other related terms, see Additional File 2). As opposed to the pre-processing done prior to the classification experiments, we did not remove Dutch stop words, since these often contain intensifiers (e.g. *nl:”heel” | en: “very”*) and negators (e.g. *nl:“niet” | en:”not”, nl:“geen” | en:”no/none”*) that impact the calculation of the sentiment score. To calculate a sentiment score for each instance, we relied on four sentiment lexicons for Dutch, including the Pattern lexicon composed of 3223 qualitative adjectives, an in-house sentiment lexicon with size *n* = 434 composed of manual review annotations, the Duoman lexicon composed of 8757 word forms and the NRC Hashtag Lexicon including 13,683 entries [30–32]. The original lexicon of 14,182 unigrams, which had been automatically translated to Dutch, was manually filtered to improve its quality. In addition, all lexicons were manually checked to filter irrelevant entries. The order in which these lexicons were consulted was determined by preliminary experiments (i.e. when a word had no match in the Pattern lexicon, the next step was to consult the in-house lexicon, followed by Duoman, and finally NRC). Sentiment scores per instance were calculated based on the sum of the retrieved sentiment word tokens (i.e. words with positive, negative or neutral valence according to the lexicons).

## Results

### Study population

One-hundred eighty-seven (*n* = 187) participants provided written informed consent, of which 121 patients with a diagnosis of migraine (*n* = 81) or CH (*n* = 40) provided a digitally written description on their headache disorder. All other participants did not provide data.Thus, the main corpus was developed with 121 full texts. After manual thematic annotation of all texts, a second corpus containing only attack descriptions was developed featuring 112 texts from 112 participants. This corpus was then used for the ML experiments on headache disorder classification. Demographic characteristics and textual characteristics of the study population and both corpora are found in Table 1.

**Table 1:** Demographic characteristics and textual characteristics for the main corpus and corpus with headache attack descriptions

	**Corpus with full texts**			**Corpus with headache attack descriptions only**		
	**All patients**	**Migraine**	**Cluster headache**	**All patients**	**Migraine**	**Cluster headache**
**Number**	121	81	40	112	74	38
**Mean age in years (SD)**	45 (13)	43.1 (12)	48.9 (14.2)	45 (13)	42 (12)	50 (13.6)
**Number of females (percentage)**	72 (60%)	64 (80%)	8 (20%)	68 (60.7%)	61 (82.4%)	7 (18.4%)
**Tokens per text: median (Q1-Q3)**	476 (218–765)	474 (227–745)	508 (198–794)	156 (80–242)	152 (84–242)	156 (71–223)
**Types per text: median (Q1-Q3)**	231 (130–321)	224 (131–317)	236 (126–341)	94 (60–131)	89 (61–133)	96 (55–122)
**Sentences per text: median (Q1-Q3)**	23 (10–42)	23 (11–41)	22 (8–40)	7 (3–12)	8 (4–12)	5 (2–11)

*Legend*: *Q1* Lower quartile, *Q3* Upper quartile, *SD* Standard deviation

### Thematic analysis of full texts

The proportion of themes in the full text patient narratives was highest for attack descriptions (median 29.5%), past treatment efforts (median 17.5%), patient medical history (median 11.3%) and burden of disease (median 11.9%). There were no significant differences in terms of proportions per theme in the full texts by migraine and CH patients. An overview of the distribution of proportions in texts for each theme can be found in Table 2.

**Table 2:** Thematic analysis from full texts (median proportions per text with first and third quartiles)

	**Full cohort**	**Migraine**	**Cluster headache**
**Attack description**	29.5% (15.8%-46.6%)	28.4% (16.0%-45.3%)	34.3% (15.8%-49.7%)
**Treatment**	17.5% (4.8%-29.2%)	15.2% (4.6%-26.9%)	20.5% (7.8%-30.3%)
**Patient medical history**	11.3% (3.9%-24.8%)	11.3% (3.9%-28.7%)	12.7% (4.2%-23.4%)
**Burden of disease**	11.9% (0%-25.7%)	13.2% (3.9%-24.4%)	6.8% (0%-26.7%)
**Triggers**	1.4% (0%-9.3%)	2.8% (0%-9.7%)	0% (0%-4.4%)
**Comorbidities**	0% (0%-0%)	0% (0%-0%)	0% (0%-0%)
**Technical investigations**	0% (0%-0%)	0% (0%-0%)	0% (0%-0%)

### Lexical analysis of full texts

**Fig. 1:**
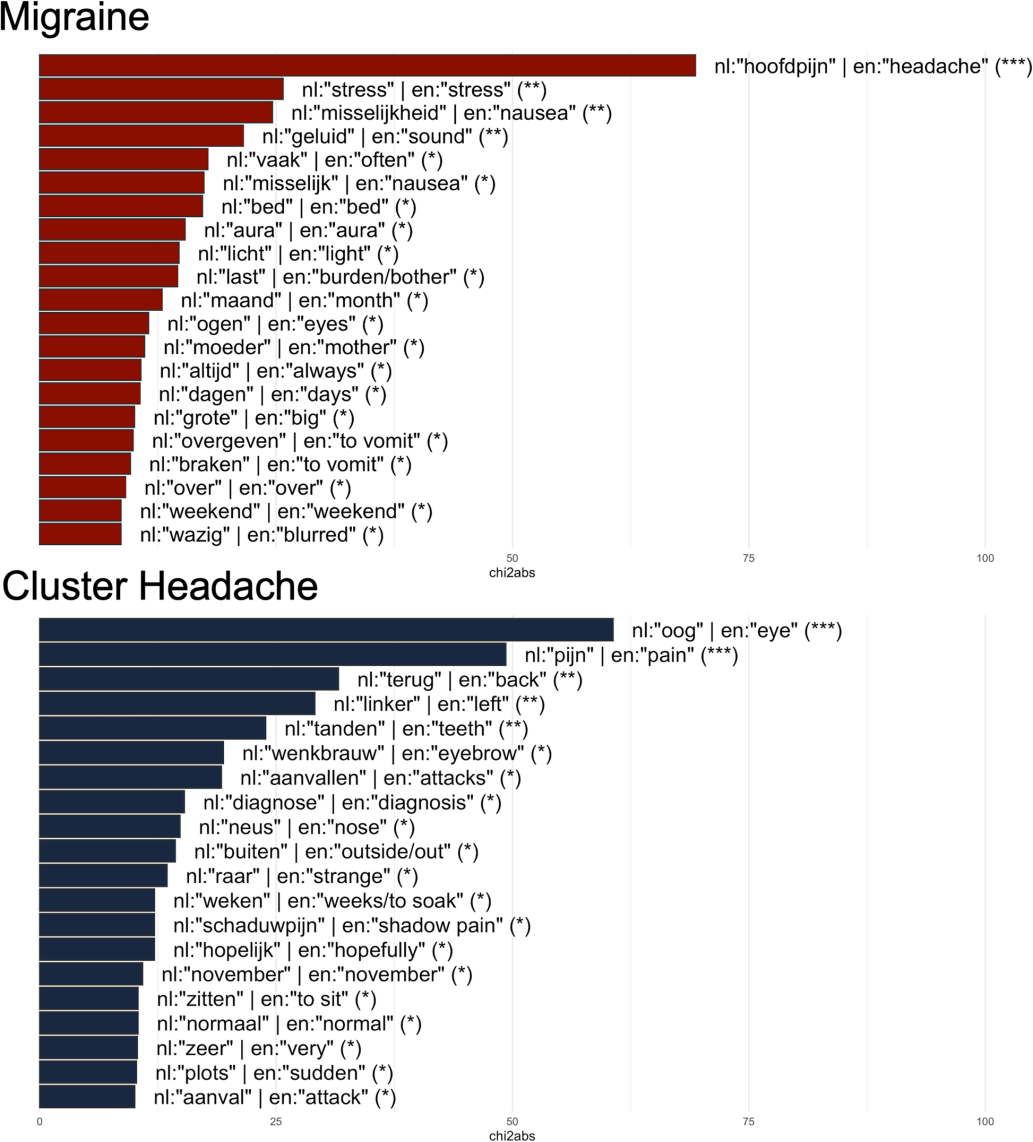
Key words per diagnosis (red colour migraine, blue colour cluster headache). Legend: (*) = *p* < 1*10^–2^, (**) = *p* < 1*10^–5^, (***) = *p* < 1*10^–8^. Abbreviations: chi2abs = absolute value of the chi-squared statistic, en = English, nl = Dutch

Distinctive word tokens between migraine and CH were highly significant for *nl:“oog” | en: “eye”*, *nl:“pijn” | en:“pain”*, *nl:“terug” | en: “back/ to come back”*, *nl: “linker” | en:“left side”* and *nl: “tanden” | en: “teeth”* for texts by participants with CH, versus *nl:“hoofdpijn” | en:“headache”*, *nl:“stress” | en:“stress”* and *nl:“misselijkheid” | en:“nausea”*, *nl:“geluid” | en:“sound”* and *nl:“vaak” | en:“often”* in texts by participants with migraine (Fig. 1 and Additional File 3).

### Lexicon-based sentiment analysis

Lexicon-based sentiment analysis for both full texts and headache attack descriptions shows that the majority of the instances express a negative sentiment (Table 3).

**Table 3:** Lexicon-based sentiment analysis statistics of the attack descriptions

Dataset	Sentiment distribution within full texts	Sentiment distribution within headache attack descriptions
All patients	86% negative (104/121), 14% positive (17/121)	96% negative (107/112), 4% positive (5/112)
Cluster headache	85% negative (34/40), 15% positive (6/40)	95% negative (36/38), 5% positive (2/38)
Migraine	86% negative (70/81), 14% positive (11/81)	96% negative (71/74), 4% positive (3/74)

When taking a closer look at the retrieved sentiment words we observed that, for the negative instances, many words referred to how patients experience their headaches and which associated symptoms are experienced (e.g. tension, tiredness, throwing up, fear, sensitivity to smells, blurred vision, et cetera).

Our qualitative analysis of the data using sentiment lexicons results in an overestimation of positive sentiment, because often positive sentiment words were recognised by the system although they were part of a negative context (e.g. en:“light” (nl: “licht”), en:“intense” (nl: “heftig”)). Frequently retrieved positive sentiment words are often part of descriptions of specific situations, coping mechanisms (e.g. resting, calm activities), or words are identified as positive while they are being used in a negative context (e.g. ‘considerable’ in ‘a considerable mistake’, ‘luckily’ as part of the sentence’luckily people who have never experienced this are not aware of it’).

### Machine learning experiments

As mentioned above, the corpus with attack descriptions with their annotated class contained 112 texts. These texts were used as input for the ML experiments.

Table 4 shows the experimental results obtained with the three classifiers, each set up with three different feature combinations as described earlier: n-gram features, metadata features (i.e. patient gender and age), and the two combined. Classification performance is presented with accuracy scores, together with precision, recall and their harmonic mean (F1-score). For the two-class classification experiments, F1-scores are micro-averaged (i.e. each label is given equal weight in the scoring).

**Table 4:** Experimental results for multi-class classification and cluster headache class detection

Two Classes													
	**Naive Bayes Classifier**				**Support Vector Machine**					**Logistic Regression**			
	**P**	**R**	**F1-score**	**Accuracy**	**P**	**R**	**F1-score**		**Accuracy**	**P**	**R**	**F1-score**	**Accuracy**
**N-grams**	0,744	0,7	0,688	0,732	0,854	0,832	0,838		**0,858**	0,856	0,83	0,838	**0,858**
**Metadata**	0,802	0,816	0,808	0,821	0,804	0,816	0,808		0,821	0,804	0,816	0,808	0,821
**N-grams + metadata**	0,744	0,7	0,688	0,732	0,838	0,826	0,828		0,849	0,848	0,84	0,84	**0,858**
**Class: cluster headache**													
	**Naive Bayes Classifier**			**Support Vector Machine**				**Logistic Regression**					
	**P**	**R**	**F1-score**	**P**	**R**	**F1-score**		**P**	**R**	**F1-score**			
**N-grams**	0,676	0,586	0,586	0,834	0,74	0,778		0,838	0,74	0,779			
**Metadata**	0,71	0,81	0,754	0,71	0,81	0,754		0,71	0,81	0,754			
**N-grams + metadata**	0,676	0,586	0,586	0,792	0,76	0,769		0,808	0,784	0,79			

Legend: Highest accuracy scores for the two classes are boldfaced, the best F1-score for the ‘cluster headache’ class is underlined. *Abbreviations: Avg* Average, *P* Precision, *R* Recall

For every fold, hyperparameter optimization was done using a grid search. For the SVM and NB classifiers, the regularization parameter C varied between 0.1 and 100 across folds. A linear kernel was used with the SVM algorithm. The class weights were balanced for each of the three algorithms.

The results demonstrate that the LR and SVM algorithms performed best for all different feature combinations compared to NB algorithms. We replicated the experimental setups with SVM and LR with n-gram features in a leave-one-out-cross-validation setup (Table 5). Also here, LR had the best results in discriminating attack descriptions by patients with migraine versus patients with CH.

**Table 5:** Experimental results for the best classifiers (SVM and LR) using leave-one-out cross-validation with n-grams features only

Two classes				
	**P**	**R**	**F1-score**	**Accuracy**
**Logistic Regression**	0,86	0,85	0,86	**0,88**
**Support Vector Machine**	0,82	0,79	0,8	0,83
**Class: cluster headache**				
	**P**	**R**	**F1-score**	
**Logistic Regression**	0,83	0,79	0,81	
**Support Vector Machine**	0,79	0,68	0,73	

Legend: Highest accuracy score for the two classes is boldfaced. The best F1-score for the ‘cluster headache’ class is underlined. *Abbreviations*: *Avg* Average, *P* Precision, *R* Recall

## Discussion

To our knowledge this is the first prospective study applying ML and NLP on written narratives containing personal experiences of headache disorders by patients with migraine or CH. Personal narratives provided by the participants contain valuable information on the characteristics of headache attacks and the burden of these disorders on patient lives [33].

For the most part, the participants with migraine or CH wrote about the most bothersome aspects of their headache disorders. The texts are largely filled with negative sentiment as shown by the multiple sentiment lexicon-based analyses of the texts. We learned that patients mostly aim to communicate descriptions of their headache attacks, their past treatment efforts, medical histories and headache burden as eloquently as possible. The written narratives were often lengthy, illustrating that patients feel the need to communicate on many aspects of their condition. Lack of information on comorbidities, triggers and technical investigations may have occurred because of the non-specific formulation of the question at the beginning of the study, which was used to create the free-writing format [33].

By looking at the lexical choices made by participants, some clear discerning patterns of word choices between patients with migraine and CH are found. Most results are in line with our scientific and clinical knowledge of the differences between both conditions. Key words per diagnosis are also highly concordant with words and phrasings within ICHD-3 diagnostic criteria [3]. A remarkable but significant observation was made that our Dutch-speaking migraine patients communicate their disorder more as *nl: “hoofdpijn” | en: “headache”* versus the CH patients, who use the word *nl: “pijn” | en: “pain”* more frequently, and that this result was highly statistically significant. This observation was previously unknown to us headache experts and shows the potential of NLP to discover latent linguistic aspects in patients’ narratives. Our understanding of the finding is that it shows a linguistic nuance which may tell us something about the underlying differences in the biology, pathophysiology and semiology of the two conditions. It reflects previous research where the painful experiences of migraine and CH may theoretically be different: migraine described as a “visceral” pain disorder and CH described rather as an “exteroceptive” pain [34]. More research on larger patient cohorts will be required to confirm our finding.

Looking at the results of the ML experiments, we can conclude that the best results are obtained by SVM and LR with N-gram features, which largely outperform the score obtained by NB. When considering the positive class only (i.e. ‘cluster headache’), we conclude that LR performs slightly better than SVM with n-gram and metadata features combined. Surprisingly enough, none of the classifiers benefit from combining n-gram features with metadata features, since there is no improvement over the scores obtained with mere n-grams. As our experiments are conducted on a small dataset, it is difficult to estimate the true difficulty of the classification task. In fact, many classifiers tend to benefit from more training data so that a more generalizable model can be constructed. The results from the leave-one-out cross-validation experiments show a slight improvement over the scores in the five-fold cross-validation setup for the LR classifier, which could suggest that more training data might boost the classification performance further. However, the scores are not better for the SVM classifier. Hence, a larger-sized corpus is deemed necessary to see whether and to what extent the corpus size influences the classifier performance for our task.

At the moment, ICHD-3 applied by humans (i.e. physicians) remains the standard for the diagnosis of headache disorders. ICHD-3 has many different headache disorder categories each with a fixed set of criteria providing a large multiclass diagnostic process [4]. However, this application of a fixed set of criteria may often result in patients fulfilling a “probable” diagnosis. For example, the American Migraine Prevalence and Prevention Study (AMPP) already showed that probable migraine is a frequent and often disabling disorder [35]. Big data projects with ML and NLP may apply supervised and unsupervised analysis to overcome these limitations in a way by discerning new classes of headache patients based on direct patient-derived linguistic data of headache disorders. This new methodology may then serve to build new classification criteria in an era of digitized headache medicine which may prove beneficial for both patients and physicians.

Our study shows that NLP and ML have the potential to perform precise, accurate and unbiased analysis of patient narratives.. We were able to build models with good to very good discriminatory capabilities with only a small to medium sized cohort. Because the largest part of our methodology is language non-specific, we believe this type of research has potential to provide good results in other regions or language domains and should be investigated in the future. Our research fits within a list of previous successful NLP and ML studies within headache medicine that also reached good classification performance on patient-specific descriptions from case files or structured questionnaires [36–38]. It adds to the perspective that the application of artificial intelligence and NLP within this area may contribute to our understanding of headache disorders, also by helping to structure scientific information in the field [39].

Limitations to the study need to be addressed. First, our patient cohort was moderately large, and additional individual narratives will presumably help ML models to become more accurate in the future. This is especially true for deep learning classification algorithms, which were not used in this study due to the rather small dataset. This also applies to the sentiment analysis, which would thrive from more narratives too so that a machine learning model for sentiment analysis can be trained and hence more intricate and complex sentiment or emotion patterns be detected. Fine-grained analysis (also known as “aspect-based sentiment or emotion analysis”) could be applied to identify the object of the sentiment or emotion within a sentence (i.e. to which part of the description does a particular emotion apply?). It would be interesting to see to which extent fine-grained sentiment information enhances the performance of the diagnosis classification. Second, the methodology of our study used the free text format, where no limitations or boundaries were put forward for participants. This resulted in the acquisition of many informative aspects of their headache disorders, but also a lot of unrelated and noisy data. Looking at the results from key words per diagnostic group however, multiple words are directly informative for a certain condition. It guides the research into more focussed questions to solve specific problems. Third, the research was limited to migraine and CH as a proof-of-concept study, and did not look into other headache disorders or diagnostic categories within ICHD-3. Fourth, our study did not control for the level of education of participants. The length of texts and type of education may influence lexical and structural grammatical choices within the self-reported narratives of participants. We plan to include such features in future analyses as they may impact the results of ML experiments. Fifth, our participants all had Dutch as native language. Future studies could also include and examine narratives by non-native speakers and compare them to results from native speakers. Previous NLP experiments used document similarity measures to describe differences between native and non-native speakers of English and applied those characteristics to statistically distinguish texts between different populations of native versus non-native speakers [40]. By doing so, this information on language nativity status can be used as features for new ML experiments to improve the accuracy of classification models for both native and non-native speakers.

## Conclusion

This research reveals differences in lexical choices NLP between patients with migraine and CH which are detected with NLP. These words are congruent with domain expert knowledge of the disorders. Furthermore, we have evaluated state-of-the-art ML algorithms to accurately classify patient narratives of migraine and CH patients. Our study shows that NLP has the potential to perform more precise, accurate and unbiased analysis of patient narratives. It may serve headache medicine in unravelling deeper layers of linguistic information which may be hidden to immediate human understanding or may be computationally too demanding for humans to fully integrate into clinical practice. NLP shows its capability to discern relevant linguistics aspects in narratives by patients with different headache disorders, which may lead to better feature engineering for new ML models in the future. Future ML and NLP experiments should focus on more direct questioning to efficiently receive qualitative information on the most relevant topics such as attack descriptions or burden of disease. Since ML algorithms thrive on information, larger amounts of textual data from a higher number of patients will be necessary.

## Supplementary Information

**Additional file 1.** Information for the participant and question asked to each participants (original in Dutch, English translated version added).

**Additional file 2.** List of classification labels and class-related words that were removed from the attack description corpus during pre-processing.

**Additional file 3.** Key words per diagnosis for migraine and cluster headache) with absolute value of the chi-squared statistical test.

## Data Availability

The datasets used and/or analysed during the current study are available from the corresponding author on reasonable request.

## Abbreviations

CH: Cluster Headache
E: Experience
ICHD-3: International Classification of Headache Disorders, Third Edition
LR: Logistic Regression
ML: Machine Learning
NLP: Natural Language Processing
NB: Naive Bayes
P: Performance Measure
SVM: Support Vector Machine
T: Task
