# Supplementary material for: Using natural language processing to automatically classify written self-reported narratives by patients with migraine or cluster headache
Source: J Headache Pain. 2022 Sep 30;23(1):129. doi: 10.1186/s10194-022-01490-0 (PMC9524092; doi:10.1186/s10194-022-01490-0)
Supplement: Supplementary file 1 — Additional file 1. Information for the participant and question asked to each participants (original in Dutch, English translated version added). [file 10194_2022_1490_MOESM1_ESM.docx]

## Additional file 1: Information for the participant and question asked to each participants (original in Dutch, English translated version added)

| **Original text (Dutch)** | **Translation (English)** |
| --- | --- |
| Geachte mevrouw, Geachte heer,  U ging recent akkoord tijdens de raadpleging bij het UZ Gent om deel te nemen aan dit universitair onderzoek. Deze studie werd goedgekeurd door de Commissie voor Medische Ethiek van het Universitair Ziekenhuis Gent.  Op de volgende pagina wordt u gevraagd om een zo gedetailleerd mogelijke tekst over uw hoofdpijnproblematiek te schrijven.  U krijgt de volledige vrijheid om te schrijven over uw hoofdpijnklachten in uw eigen bewoordingen en volgens de verschillende aspecten en kenmerken van de hoofdpijn die u belangrijk vindt.  Wij vragen u vriendelijk om geen eigennamen te gebruiken in de tekst.  Klik op de knop "submit" onderaan deze pagina om naar de volgende pagina te gaan.  Wij wensen uitdrukkelijk en van harte te danken voor uw deelname aan dit academisch onderzoek.  Met vriendelijke groeten,  De onderzoekers en  UZ Gent / Universiteit Gent | Dear Madam, Sir,  You recently gave permission to participate in academic research at Ghent University Hospital. This study was approved by the Regulatory Ethics Board at Ghent University Hospital.  On the next page, you will be asked to write about your headache disorder as detailed as possible.  You will have complete freedom to write about your headache-related complaints and to describe all aspects and characteristics of headache you find important.  We kindly ask you to not use any proper names in the text.  Please click “submit” at the bottom of this page to go to the next page.  We explicitly want to thank you for your participation in this academic research.  Kind regards,  The investigators and Ghent University Hospital / Ghent University |
