# Supplementary material for: Using natural language processing to automatically classify written self-reported narratives by patients with migraine or cluster headache
Source: J Headache Pain. 2022 Sep 30;23(1):129. doi: 10.1186/s10194-022-01490-0 (PMC9524092; doi:10.1186/s10194-022-01490-0)
Supplement: Supplementary file 2 — Additional file 2. List of classification labels and class-related words that were removed from the attack description corpus during pre-processing. [file 10194_2022_1490_MOESM2_ESM.docx]

## Additional file 2: List of classification labels and class-related words that were removed from the attack description corpus during pre-processing.

| Aimovig, chp, chp-aanvallen, chp-patiënt, cluster, clusters, clusteraanval, clusterhoofdpijnaanvallen, clusterhoofdpijn, clusterhoofdpijnen, clusterperiode, cm, dafalgan, dafalgans, excedrin, excedryn, ibuprofen, imitrex, migraine, migraine-aanval, migraine-aanvallen, migraineaanval, migraineaanvallen, migrainepatiënt, migrainepijn, motilium, naratriptan, paracetamol, suma, sumatriptan, sumtriptan |
| --- |
