# Supplementary material for: Using natural language processing to automatically classify written self-reported narratives by patients with migraine or cluster headache
Source: J Headache Pain. 2022 Sep 30;23(1):129. doi: 10.1186/s10194-022-01490-0 (PMC9524092; doi:10.1186/s10194-022-01490-0)
Supplement: Supplementary file 3 — Additional file 3. Key words per diagnosis for migraine and cluster headache) with absolute value of the chi-squared statistical test. [file 10194_2022_1490_MOESM3_ESM.docx]

## Additional file 3: Key words per diagnosis for migraine and cluster headache) with absolute value of the chi-squared statistical test.

*Legend: en = English, nl = Dutch*

| **MIGRAINE** | **chi2** | **P-value** | **CLUSTER HEADACHE** | **Chi2** | **P-value** |
| --- | --- | --- | --- | --- | --- |
| nl:"hoofdpijn" \| en:"headache" | 69.34 | 1.10E-16 | nl:"oog" \| en:"eye" | 60.64 | 6.90E-15 |
| nl:"stress" \| en:"stress" | 25.74 | 3.90E-07 | nl:"pijn" \| en:"pain" | 49.29 | 2.20E-12 |
| nl:"misselijkheid" \| en:"nausea" | 24.62 | 7.00E-07 | nl:"terug" \| en:"back" | 31.59 | 1.90E-08 |
| nl:"geluid" \| en:"sound" | 21.54 | 3.50E-06 | nl:"linker" \| en:"left" | 29.1 | 6.90E-08 |
| nl:"vaak" \| en:"often" | 17.79 | 2.50E-05 | nl:"tanden" \| en:"teeth" | 23.89 | 1.00E-06 |
| nl:"misselijk" \| en:"nausea" | 17.37 | 3.10E-05 | nl:"wenkbrauw" \| en:"eyebrow" | 19.42 | 1.00E-05 |
| nl:"bed" \| en:"bed" | 17.22 | 3.30E-05 | nl:"aanvallen" \| en:"attacks" | 19.22 | 1.20E-05 |
| nl:"aura" \| en:"aura" | 15.38 | 8.80E-05 | nl:"diagnose" \| en:"diagnosis" | 15.29 | 9.20E-05 |
| nl:"licht" \| en:"light" | 14.73 | 1.20E-04 | nl:"neus" \| en:"nose" | 14.84 | 1.20E-04 |
| nl:"last" \| en:"burden/bother" | 14.59 | 1.30E-04 | nl:"buiten" \| en:"outside/out" | 14.34 | 1.50E-04 |
| nl:"maand" \| en:"month" | 12.95 | 3.20E-04 | nl:"raar" \| en:"strange" | 13.47 | 2.40E-04 |
| nl:"ogen" \| en:"eyes" | 11.52 | 6.90E-04 | nl:"weken" \| en:"weeks/to soak" | 12.14 | 4.90E-04 |
| nl:"moeder" \| en:"mother" | 11.1 | 8.60E-04 | nl:"hopelijk" \| en:"hopefully" | 12.13 | 5.00E-04 |
| nl:"altijd" \| en:"always" | 10.71 | 0.0011 | nl:"schaduwpijn" \| en:"shadow pain" | 12.13 | 5.00E-04 |
| nl:"dagen" \| en:"days" | 10.62 | 0.0011 | nl:"november" \| en:"november" | 10.88 | 9.70E-04 |
| nl:"grote" \| en:"big" | 10.04 | 0.0015 | nl:"normaal" \| en:"normal" | 10.4 | 0.0013 |
| nl:"overgeven" \| en:"to vomit" | 9.89 | 0.0017 | nl:"zitten" \| en:"to sit" | 10.4 | 0.0013 |
| nl:"braken" \| en:"to vomit" | 9.6 | 0.0019 | nl:"zeer" \| en:"very" | 10.32 | 0.0013 |
| nl:"over" \| en:"over" | 9.09 | 0.0026 | nl:"plots" \| en:"sudden" | 10.23 | 0.0014 |
| nl:"wazig" \| en:"blurred" | 8.61 | 0.0033 | nl:"aanval" \| en:"attack" | 10.05 | 0.0015 |
| nl:"weekend" \| en:"weekend" | 8.61 | 0.0033 |  |  |  |
